# Engineering and characterisation of chimeric monoclonal antibody 806 (ch806) for targeted immunotherapy of tumours expressing de2-7 EGFR or amplified EGFR

**DOI:** 10.1038/sj.bjc.6602470

**Published:** 2005-03-15

**Authors:** C Panousis, V M Rayzman, T G Johns, C Renner, Z Liu, G Cartwright, F-T Lee, D Wang, H Gan, D Cao, A Kypridis, F E Smyth, M W Brechbiel, A W Burgess, L J Old, A M Scott

**Affiliations:** 1 Ludwig Institute for Cancer Research, Melbourne Tumour Biology Branch, Level 1, Harold Stokes Building, Austin Hospital, 145-163 Studley Road, Heidelberg 3084. Victoria, Australia; 2 Radioimmune & Inorganic Chemistry Section, Radiation Oncology Branch, National Cancer Institute. Bethesda, MD 20892, USA; 3 Ludwig Institute for Cancer Research, Memorial Sloan Kettering Cancer Centre, New York, NY 10021, USA

**Keywords:** EGFR, chimeric antibody, immunotherapy, EGFRvIII

## Abstract

We report the generation of a chimeric monoclonal antibody (ch806) with specificity for an epitope on the epidermal growth factor receptor (EGFR) that is different from that targeted by all other anti-EGFR therapies. Ch806 antibody is reactive to both de2-7 and overexpressed wild-type (wt) EGFR but not native EGFR expressed in normal tissues at physiological levels. Ch806 was stably expressed in CHO (DHFR −/−) cells and purified for subsequent characterisation and validated for use in preliminary immunotherapy investigations. Ch806 retained the antigen binding specificity and affinity of the murine parental antibody. Furthermore, ch806 displayed enhanced antibody-dependent cellular cytotoxicity against target cells expressing the 806 antigen in the presence of human effector cells. Ch806 was successfully radiolabelled with both iodine-125 and indium-111 without loss of antigen binding affinity or specificity. The radioimmunoconjugates were stable in the presence of human serum at 37°C for up to 9 days and displayed a terminal half-life (*T*_1/2β_) of approximately 78 h in nude mice. Biodistribution studies undertaken in BALB/c nude mice bearing de2-7 EGFR-expressing or amplified EGFR-expressing xenografts revealed that ^125^I-labelled ch806 failed to display any significant tumour retention. However, specific and prolonged tumour localisation of' ^111^In-labelled ch806 was demonstrated with uptake of 31%ID g^−1^ and a tumour to blood ratio of 5 : 1 observed at 7 days postinjection. *In vivo* therapy studies with ch806 demonstrated significant antitumour effects on established de2-7 EGFR xenografts in BALB/c nude mice compared to control, and both murine 806 and the anti-EGFR 528 antibodies. These results support a potential therapeutic role of ch806 in the treatment of suitable EGFR-expressing tumours, and warrants further investigation of the potential of ch806 as a therapeutic agent.

Overexpression of the epidermal growth factor receptor (EGFR) has been observed in many tumours including the breast, lung, colon, prostate, head and neck, and brain, and increased EGFR expression frequently correlates with more aggressive clinical course (Nicholson *et al*, 2001; Arteaga, 2002; Mendelsohn, 2002). This overexpression of the receptor is commonly the result of the *EGFR* gene amplification (Hendler and Ozanne, 1984; Sainsbury *et al*, 1987; Salomon *et al*, 1995). *EGFR* gene amplification and subsequent overexpression of the EGFR protein is particularly prevalent in gliomas, the most common primary tumour of the central nervous system (Wikstrand *et al*, 1997). Indeed, the highly malignant glioblastoma multiforme exhibits *EGFR* gene amplification at a frequency of 40–50%, with many tumours also exhibiting structural rearrangements of the *EGFR* (Voldborg *et al*, 1997). The most common of these variant *EGFR* genes contains an in-frame 801 bp deletion that removes exons 2–7 of the *EGFR* gene (Sugawa *et al*, 1990; Wong *et al*, 1992; Frederick *et al*, 2000). This resulting receptor, known as the de2-7 EGFR, does not bind ligand but displays a low level of constitutive activity and imparts a significant *in vivo* growth advantage to a number of tumour types including the breast, lung and particularly gliomas (Nishikawa *et al*, 1994; Wikstrand *et al*, 1998; Tang *et al*, 2000).

Because the EGFR is overexpressed in many types of cancer, and its blockade often inhibits tumour growth, this receptor is a rational target for novel cancer therapeutics. Potential anti-EGFR therapeutics include anti-EGFR antibodies (Sato *et al*, 1987), EGFR-ligand conjugates, EGFR immunoconjugates, small molecular weight tyrosine kinase inhibitors (Fry *et al*, 1994; Levitzki and Gazit, 1995), dominant-negative EGFR constructs, antisense EGFR oligonucleotides, all of which are capable of blocking EGFR function (de Bono and Rowinsky, 2002; Herbst and Langer, 2002; Herbst and Shin, 2002; Wakeling, 2002).

A number of antibodies directed to the extracellular domain of the EGFR have been tested in the clinic including EMD 55900 (Bier *et al*, 2001), ABX-EGF (Yang *et al*, 2001) and IMC-C225 (Mendelsohn, 1997) with some antitumour activity shown in patients. The most clinically advanced of these is IMC-225, that is currently in Phase II/III clinical trials and has recently been approved by the FDA for use in conjunction with irinotecan in patients with irinotecan refractory colorectal cancer (Stragliotto *et al*, 1996; Herbst and Langer, 2002; Lynch and Yang, 2002). Significant uptake in normal tissue such as the liver and skin is a potential limitation of antibodies targeting the wild-type (wt) EGFR (Divgi *et al*, 1991; Busam *et al*, 2001). While targeting of normal tissue usually produces manageable side effects such as skin rash, it does mean these antibodies are inappropriate for coupling with cytotoxic agents or radioisotopes.

Several antibodies have also been developed that specifically target the tumour-specific de2-7 EGFR (Humphrey *et al*, 1990; Hills *et al*, 1995; Wikstrand *et al*, 1995). These antibodies are directed to the unique junctional peptide formed by the deletion event in the de2-7 EGFR and therefore are specific for this receptor and do not bind the wt EGFR. We recently described a novel monoclonal antibody (mAb 806) generated by immunising mice with cells expressing the full-length de2-7 EGFR (Luwor *et al*, 2001; Mishima *et al*, 2001; Johns *et al*, 2002; Jungbluth *et al*, 2003). Scatchard analysis demonstrated mAb 806 binds only approximately 10% of the wt EGFR on the surface of A431 cells, a cell line that over-expresses the wt EGFR (Johns *et al*, 2002). In the absence of ligand, the majority of wt EGFR on the cell surface is in an inactive or ‘tethered’ form. In order to form active dimers, the EGFR untethers and undergoes a conformational change. The mAb 806 epitope is only exposed when the receptor is in this transient state between inactive and active dimer (Johns *et al*, 2004). Thus, at any given time point, mAb 806 can only bind this small fraction of EGFR in the correct conformation. Interestingly, untethering appears to occur at an increased rate in cells overexpressing EGFR, possibly due to changes in glycosylation (Johns *et al*, 2004). The extracellular deletion associated with the de2-7 EGFR appears to expose the mAb 806 epitope allowing constitutive binding of the antibody. The epitope is different from that targeted by all other anti-EGFR therapies and is not accessible in normal human tissues (Jungbluth *et al*, 2003). Studies with mAb 806 in nude mice have shown significant antitumour activity against xenografts expressing de2-7 EGFR or amplified EGFR, independent of antibody effector function (Johns *et al*, 2002, 2003). Given the unusual specificity profile and direct antitumour activity, we undertook to develop this anti-EGFR antibody as a construct with minimal immunogenicity for potential use in clinical trials. Herein we describe the construction and subsequent *in vitro* and *in vivo* characterisation of a chimeric mouse–human IgGl construct of mAb 806 (ch806).

## MATERIALS AND METHODS

### Antibodies and cell lines

The murine mAb 806 was generated following the immunisation of mice with NR6 mouse fibroblasts expressing the de2-7 EGFR (Jungbluth *et al*, 2003). The 528 antibody, which recognises both de2-7 and wt EGFR, has been previously described (Luwor *et al*, 2001).

The dihydrofolate reductase (DHFR)-deficient CHO cell line DG44 was obtained from Kyowa Hakko Kogyo Co. Ltd, Japan, with the permission of Professor Chasin, Columbia University, USA (Morris *et al*, 1990). These CHO cells were cultured in IMDM supplemented with 10% fetal calf serum (FCS, CSL Ltd, Parkville, Victoria, Australia) and hypoxanthine–thymidine (Invitrogen Life Technologies, Melbourne, Victoria, Australia).

The human glioblastoma cell line U87MG, expressing the wt EGFR endogenously, and the transfected cell lines U87MG.de2-7, U87MG.wtEGFR expressing the de2-7 EGFR and overexpressing the wt EGFR, respectively, have been described previously (Nishikawa *et al*, 1994; Nagane *et al*, 1996). The human epidermoid carcinoma-derived cell line (A431) has also been described (Sato *et al*, 1987). U87MG cell lines and A431 cells were cultured as previously described (Luwor *et al*, 2001). A431 cells express 2 × 10^6^ EGFR/cell through amplification of *EGFR* gene. The 806 antibody is capable of binding only approximately 10% of the EGFR present on the A431 cell surface (Johns *et al*, 2002). The FaDu (HTB-43) squamous cell carcinoma was obtained from ATCC (Manassas, VA, USA). This cell line displays normal cell expression levels of EGFR (2 × 10^5^ EGFR/cell). FaDu cells were maintained in DMEM (DMEM/F12; Invitrogen Life Technologies, Grand Island, NY, USA) containing 10% FCS, 2 mM glutamine (Sigma Chemical Co., St Louis, MO, USA), and penicillin/streptomycin (Invitrogen Life Technologies, Melbourne, Victoria, Australia). The 806 antibody shows negligible binding to these cells by FACS analysis (data not shown).

### Sequencing of murine 806 variable antibody regions

Total cytoplasmic RNA from the 806 hybridoma was obtained using standard RNA isolation techniques (Chomczynski and Sacchi, 1987). The first strand cDNA synthesis kit (Amersham Pharmacia Biotech, Castle Hill, NSW, Australia) was employed to generate the single strand cDNA. Control cDNA synthesis reactions were carried out in the absence of reverse transcriptase. PCR was carried out on 0.2 pmol *μ*l^−1^ of the cDNA template, 25 pmol *μ*l^−1^ primers in the presence of 2.5 units of Platinum *Taq* DNA polymerase High Fidelity (Invitrogen Life Technologies, Melbourne, Victoria, Australia) under standard conditions (60 mM Tris-SO_4_, pH 8.9, 18 mM (NH_4_)_2_SO_4_, 2 mM MgSO_4_, 0.2 mM of each dNTP) in volumes of 50 *μ*l in MJ Research PTC200 thermocycler (GeneWorks, SA, Australia). Primers for the amplification of heavy and light chains were, in the case of 5′-end primers, a compilation based on the leader peptide sequence of known mouse antibodies (Jones and Bendig, 1991). In regard to the 3′-end primers, the light-chain primer hybridised within the mouse kappa constant region not too distant from the V–C junction. The heavy-chain primer hybridised within the CHI constant region not far from the V–CH1 junction. In all cases, 30 cycles of amplification were carried out following the ‘hot-start’ using the following parameters: denaturation for 1 min, 94°C; annealing 1 min 45–65°C; extension, 2 min at 68°C. A final 10-min extension step at 68°C followed the 30 cycles. The PCR products were analysed by DNA electrophoresis, cloned using the TA Cloning System (Invitrogen Life Technologies, Melbourne, Australia) then extensively sequenced. Pseudogenes that were cloned during this procedure were identified following sequencing and eliminated from the further analysis. Novel VH and VL regions were identified for the 806 antibody and used in the subsequent chimerisation strategy.

### Construction of a mouse–human chimeric 806 antibody

The ch806 antibody was designed to have the variable regions of heavy and light chains of murine 806 linked to human gamma-1 and kappa constant regions, respectively. PCR primers used to modify the 5′- and 3^′^-sequences flanking the cDNA sequences coding for the mouse 806 VL and VH regions incorporated Kozak sequence (Kozak, 1987) and *Pme*l restriction enzyme site (for 5′ primers), and splice donor site and *Bam*HI restriction enzyme site (for 3′ primers). The sequences of the primers were as follows: VL 5′-end primer AGCTTTGTTTAAACGCCGCCACCATGGTGTCCACAGCTCAGTTCC; VL 3′-end primer CCGAGGATCCACTCACGTTTGATTTCCAGCTTGGTGCC; VH 5′-end primer AGCTTTGTTTAAACGCCGCCACCATGAGAGTGCTGATTCTTTTGTGG; VH 3′-end primer CCGAGGATCCACTCACCTGCAGAGACAGTGACCAGAGT. The adapted amplified mouse 806 variable regions were subsequently subcloned into mammalian cell expression vectors already containing the human kappa (pREN-Neo vector) or gamma-1 (pREN-DHFR vector) constant regions. The vectors employ parts of the human elongation factor promoter/enhancer sequence (Mizushima and Nagata, 1990) to efficiently transcribe the light and heavy chains, and provide high-level antibody production via a mechanism of internal translation initiation in bicistronic mRNA.

### Expression of ch806 antibody in DHFR-deficient CHO DG44 cells

DNA for transfections was purified from *Escherichia coli* cells using Qiagen Plasmid Midi Kit (Qiagen, Clifton Hill, Victoria, Australia) as recommended by the manufacturer. All DNA preparations were examined by restriction enzyme digestion. Sequencing of the 806 variable regions was performed at MicroMon DNA Sequencing Facility (Department of Microbiology, Monash University, Victoria, Australia). For transfection of the DHFR-deficient CHO DG44 cells, plasmids encoding heavy and light chains of the ch806 antibody (10 *μ*g circular plasmid for each construct) were cotransfected into CHO DG44 cells growing at log phase using electroporation (270 V, 975 *μ*F; BioRad, Victoria, Australia). Cells were plated in 10-cm dishes and cultured with standard medium. After 24 h, medium was replaced by fresh IMDM medium supplemented with 10% dialysed FCS (Life Technologies, Grand Island, NY), 500 *μ*g/ml Geneticin and 5 nM GMP-grade methotrexate (Life Technologies, Grand Island, NY, USA). After the initial phase of cell killing was over (days 10–14), outgrowing colonies were picked and screened for antibody production. The best producing clones were then subjected to an amplification procedure by increasing the methotrexate concentration to 100 nM and maintaining all other conditions. Outgrowing colonies under these conditions were then picked, screened and the highest producing clones taken for further analysis.

### Analysis of ch806 binding activity

FACS and BIAcore binding specificity and affinity studies were undertaken as described previously (Liu *et al*, 2003). Biacore analyses utilised recombinant soluble extracellular domain of the EGFR (sEGFR621) (Liu *et al*, 2003). For both studies, ch806 was purified from cell culture supernatant using standard recombinant Protein-A affinity and size exclusion chromatography purification. Protein concentration was measured spectrophotometrically.

### Analysis of ch806 immunological effector functions

Complement-dependent cytotoxicity (CDC) and antibody-dependent cellular cytotoxicity (ADCC) of mAb 806, ch806 and isotype control cG250 were undertaken as described previously (Scott *et al*, 2000) and utilised U87MG.de2-7 and A431 cells as target cells (effector: target cell ratio=50 : 1) over the antibody concentration range 0.00315–10 *μ*g ml^−1^. Freshly isolated PBMC effector cells for ADCC analyses and complement for the CDC assays was freshly prepared from healthy donors as described previously (Scott *et al*, 2000).

### Radiolabelling and quality assurance

Ch806 and isotype control antibody (huA33) were radiolabelled either directly with iodine-125 (^125^I) or via a bifunctional metal ion chelating agent, CHX-A″-DTPA, for indium-111 (^111^In) (Lee *et al*, 2001). Radioiodination with ^125^I (NEN Life Science Products, Boston, MA, USA) was performed via a modified chloramine-T reaction (Hunter and Greenwood, 1962), using a chloramine T : protein ratio of 3 : 1 as previously described (Lee *et al*, 2001). Antibody labelling with ^111^In was achieved via a bifunctional metal ion chelating agent, CHX-A″-DTPA (Wu *et al*, 1997) as previously described (Lee *et al*, 2001). Radiolabelling was performed on the day of injection into mice. Prior to injection, percentage (%) of unbound radionuclide content was determined by instant thin layer chromatography (ITLC), and binding ability of the final radiolabelled product was tested by a de2-7EGFR-positive cell binding (Lindmo) assay as detailed below. Scatchard analysis was used to determine the binding constant (*K*_*a*_) and number of antibody molecules bound per cell for ^125^I- and ^111^In-labelled antibody.

### Radiochemical purity

The amount of free *vs* bound antibody following radiolabelling was determined by ITLC as previously described (Lee *et al*, 2001). Assays were performed in duplicate. The percentage of isotope bound to ch806 was >90% in all experiments detailed.

### Immunoreactivity

The immunoreactive fraction of the radiolabelled constructs with U87MG.de2-7 target cells was determined by linear extrapolation to binding at infinite antigen excess using a ‘Lindmo’ assay (Lindmo *et al*, 1984) as previously described (Lee *et al*, 2001).

### Serum stability

Serum stability was assessed by incubating 5.0 *μ*g of each radiolabelled protein in 200 *μ*l of healthy donor human serum at 37°C for a 7-day period. Single-point immunoreactivity assays at 0 (no incubation), 4.0 and 7.0 days of incubation were undertaken.

### Animal models

*In vivo* studies were performed in 5–6-week-old female athymic BALB/c nude mice, homozygous for the nu/nu allele, bred by the SPF Facility, University of South Australia. Mice were maintained in autoclaved micro-isolator cages housed in a positive pressure containment rack (Thoren Caging Systems Inc., Hazelton, PA, USA). All animal studies were approved by the Austin Hospital Animal Ethics Committee and were conducted in compliance with NHMRC/CSIRO/AAC Australian Code of Practice for the Care and Use of Animals for Scientific Purposes.

To establish xenografts, mice were injected subcutaneously into the left inguinal mammary line with 3 × 10^6^ U87MG.de2-7 human glioma cells, or 5 × 10^6^ A431 adenocarcinoma cells or 5 × 10^6^ FaDu (HTB-43) control squamous cell carcinoma cells in 100 *μ*l of PBS. Tumour volume (TV) was calculated by the formula [(length × width^2^)/2] (Clarke *et al*, 2000), where length was the longest axis and width the measurement at right angles to length.

In an initial biodistribution experiment, 40 BALB/c nude mice with established U87MG.de2-7 xenografts (mean±s.d. TV=380.4±170.9 mm^3^) received radiolabelled ^111^In-ch806/^125^I-ch806 (3 *μ*g, 10 *μ*Ci ^111^In-CHX-A″-DTPA-ch806; 3 *μ*g, 3.8 *μ*Ci ^125^I-ch806) intravenously via the tail vein (total volume=0.1 ml). In a separate study, groups of 12 mice with established A431 xenograft (mean±s.d. TV=99.3±34.0 mm^3^) or established FaDu xenografts (mean±s.d. TV=134.8±33.2 mm^3^) received radiolabelled ^111^ln-ch806/^125^I-ch806 (3.3 *μ*g, 7.5 *μ*Ci ^111^In-CHX-A″-DTPA-ch806; 3.3 *μ*g, 3.8 *μ*Ci ^125^I-ch806) intravenously via the tail vein (total volume=0.1 ml). At designated time points after injection of the radioconjugates (*t*=4 h, days 1, 2, 3, 5 and 7), groups of mice (*n*=3–5) were killed by Ethrane anaesthesia. Mice were then exsanguinated by cardiac puncture, and tumours and organs (liver, spleen, kidney, muscle, skin, bone (femur), lungs, heart, stomach, brain, small bowel, tail and colon) were resected immediately. All samples were counted in a dual gamma scintillation counter (Packard Instruments). Triplicate standards prepared from the injected material were counted at each time point with tissue and tumour samples enabling calculations to be corrected for the physical decay of the isotopes. The tissue distribution data were calculated as the mean±s.d. percent injected dose per gram tissue (%ID g^−1^) for the ch806 antibody per time point.

### Pharmacokinetics

Serum obtained from mice bearing U87MG.de2-7 xenografts, following infusion of radiolabelled-ch806 as described above, was aliquoted in duplicate and counted in a gamma scintillation counter (Packard Instruments, Melbourne, Australia). Triplicate standards prepared from the injected material were counted at each time point with serum samples to enable calculations to be corrected for the isotope physical decay. The results of the serum were expressed as % injected dose per litre (% ID l^−1^).

Pharmacokinetic calculations were performed of serum data using a curve fitting program (WinNonlin, Pharsight Co., Mountain View, CA, USA). A two-compartment model was used to calculate serum pharmacokinetic parameters of AUC (area under the serum concentration curve extrapolated to infinite time), CL (total serum clearance), *T*_1/2α_ and T_1/2β_ (half-lives of the initial and terminal phases of disposition) for ^125^I- and ^111^In-ch806.

### Therapeutic *in vivo* studies

U87MG.de2-7 tumour cells (3 × 10^6^) in 100 *μ*l of media were inoculated subcutaneously (s.c.) into both flanks of 4–6-week-old female nude mice (*n*=5 group^−1^). Antibody treatment commenced day 7 post-tumour cell inoculations (mean±s.e. tumour volume=60±15 mm^3^) and consisted of six intraperitoneal (i.p.) injections over 2 weeks of 1 mg ch806, 1 mg murine 806, 1 mg murine 528 pan anti-EGFR antibody, or vehicle control. Tumour volume in mm^3^ was determined as described previously. Data were expressed as mean tumour volume±s.e. for each treatment group. Differences in tumour size between control and test groups were tested for statistical significance (*P*<0.05) by *t*-test.

## RESULTS

### Engineering and expression of ch806

Chimeric 806 cDNA was initially engineered into expression vectors where the antibody genes, and the genes used for resistance to selection, were driven by CMV and SV40 promoters, respectively. Using these expression vectors, a gradual decrease in the production rates for ch806 by transfected CHO DG44 cells was observed with increasing cell passage number. Northern analysis of clones at various passage number revealed a loss of mRNA for the expression of the heavy-chain antibody gene with no loss in message levels for either the light-chain (neomycin) or heavy-chain (DHFR) selectable markers, nor the light-chain antibody gene (data not shown).

Based on these results, a next generation expression vector system (pREN) was constructed with the engineering of an internal ribosome entry site (IRES) between the antibody genes and the selectable markers. In this system, antibody producing CHO DG44 clones cannot express the genes required for resistance to selection without expression of the antibody genes. Using the pREN vectors, all clones growing under selection conditions for light-chain expression (G418) and heavy-chain expression (methotrexate) were shown to stably produce ch806 over time.

### Binding specificity and affinity

**Figure 1:**
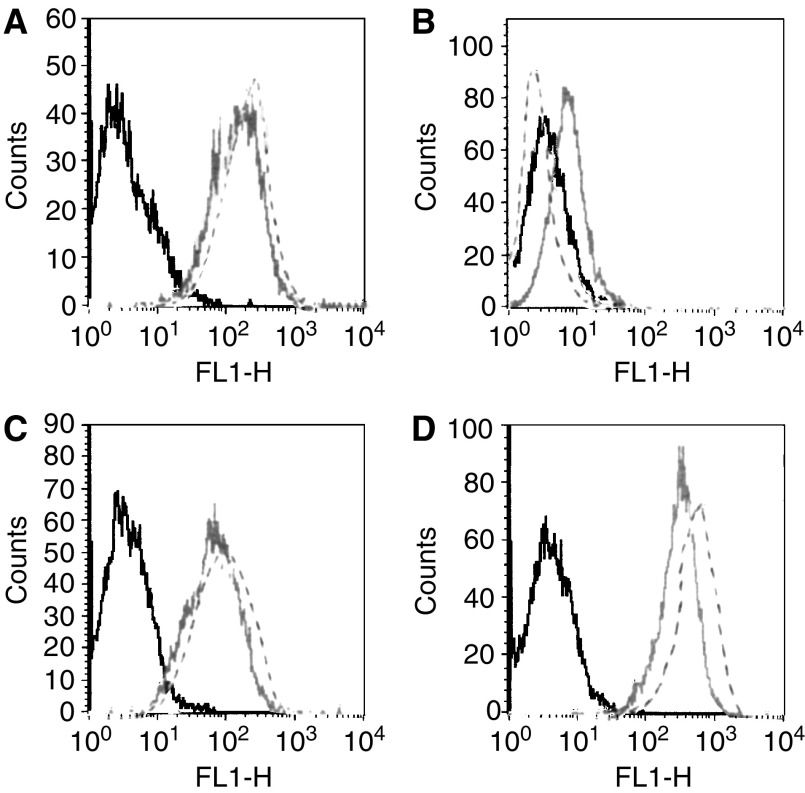
FACS analysis of (**A**) A431 adenocarcinoma cell line (amplified *EGFR)* and (**B**) parental and (**C**) transfected U87MG glioma cell lines stably expressing wt (U87MG.wtEGFR) or (**D**) mutant EGFR (U87MG.de2-7). Cells were incubated with mAb806 (–), ch806 (- -) followed by Alexa488-labelled anti-mouse Ig. The plots represent fluorescence intensity on the abscissa and cell number per fluorescence channel on the ordinate. The negative control (irrelevant antibody) fluorescence is plotted on each panel (black line).

Ch806 was compared to its murine parental antibody for binding specificity to a variety of cells expressing both mutant de2-7 EGFR and overexpressed EGFR (Figure 1). These FACS results clearly show that the ch806 antibody displays the identical binding specificity as the murine parent. As previously reported for mAb 806 (Mishima *et al*, 2001; Johns *et al*, 2002) ch806 reacted intensively with U87MG.de2-7 cells, less binding was observed with A431 cells containing amplified *EGFR*, and weak binding with U87MG.wtEGFRcells. BIAcore analysis with recombinant sEGFR revealed the chimeric antibody to have retained the entire original binding affinity of the murine parent (1.1 × 10^9^ M^−1^; data not shown).

### Immune effector functions

**Figure 2:**
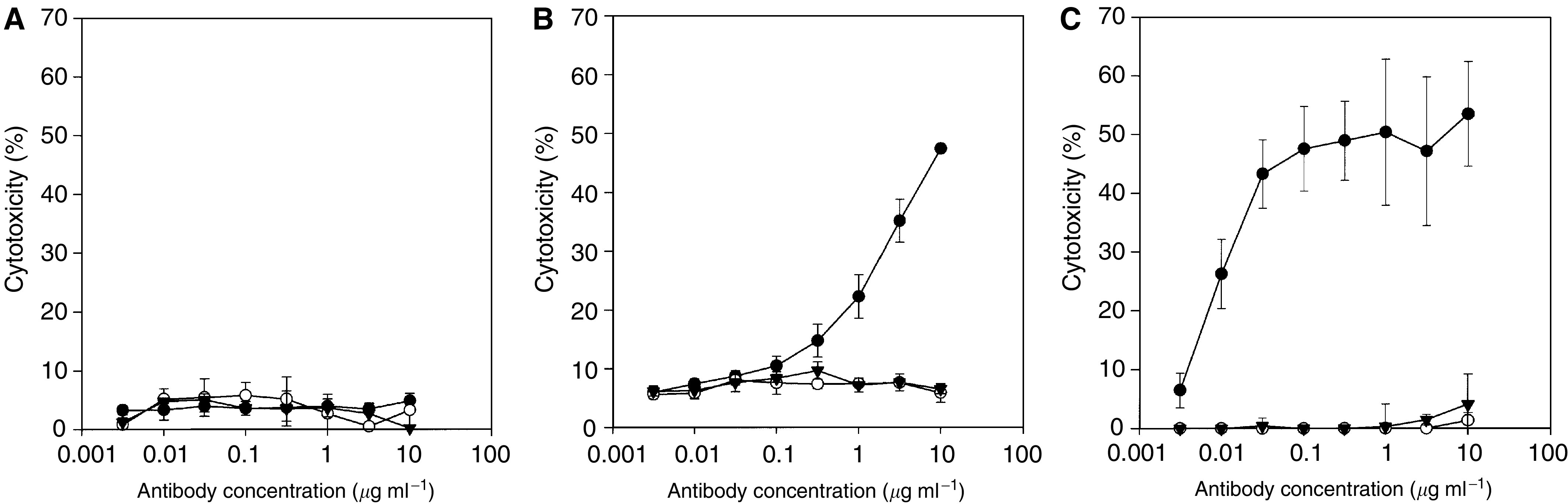
Immune effector function of mAb 806 (○), chimeric IgGl antibody ch806 (•) and Isotype control cG250 (▾). (**A**) Complement-dependent cytotoxicity activity of 0–10 *μ*g ml^−1^ antibody on target U87MG.de2-7 cells. Antibody-dependent cellular cytotoxity on target (**B**) A431 cells and (**C**) U87MG.de2-7 cells at effector-to-target cell ratio of 50 : 1 and antibody concentrations ranging from 0 to 10 *μ*g ml^−1^. Mean (bars;±s.d.) percent cytotoxicity of triplicate determinations are presented. Representative results from three separate experiments are shown.

The results of the CDC analyses are presented in Figure 2A. Minimal CDC activity was observed in the presence of up to 10 *μ*g ml^−1^ ch806 with CDC comparable to that observed with the murine mAb 806 and isotype control cG250. Ch806-mediated ADCC was determined at an E : T ratio of 50 : 1 on target A431 (Figure 2B) and U87MG.de2-7 cells (Figure 2C). In contrast to the parental mAb 806 and isotype control antibodies, high-specific cytotoxicity was observed with ch806 against target U87MG.de2-7 cells with 47% cytotoxicity effected by 0.03 *μ*g ml^−1^ ch806. Lower ADCC was mediated by ch806 on A431 cells that contain lower numbers of binding sites (Figure 2B).

### Antibody radiolabelling

Radiochemical purity assessment of radiolabelled antibody by ITLC prior to injection confirmed >98.6% bound ^125^I- (1.4% free) and >99.3% bound ^111^In-CHX-A″-DTPA. The immunoreactivity of ^l25^I-ch806 was 68.1% and ^111^In-ch806 was 69.5% for U87MG.de2-7 cells. *K*_*a*_ values for ^111^In and ^125^I were 1.36 × 10^9^ M^−1^ and 1.90 × 10^9^ M^−1^, respectively, which is highly comparable to that of the parental murine mAb806 of 1.1 × 10^9^ M^−1^ (Johns *et al*, 2002) and the BIAcore determinations. The number of antibody molecules bound per U87MG.de2-7 cell for each isotope were 6 × 10^5^ for ^111^In, and 4.7 × 10^5^ for ^I25^I.

### Serum stability

In order to assess the potential stability of ch806 administered to patients, serum stability studies were undertaken (Table 1). These results demonstrate that in the presence of human serum at 37°C for up to 7 days incubation, ch806 shows excellent stability, as measured by the radiochemical purity and percentage of antibody binding to de2-7 EGFR-expressing cells in a single-point immunoreactivity assay.

### Biodistribution

**Figure 3:**
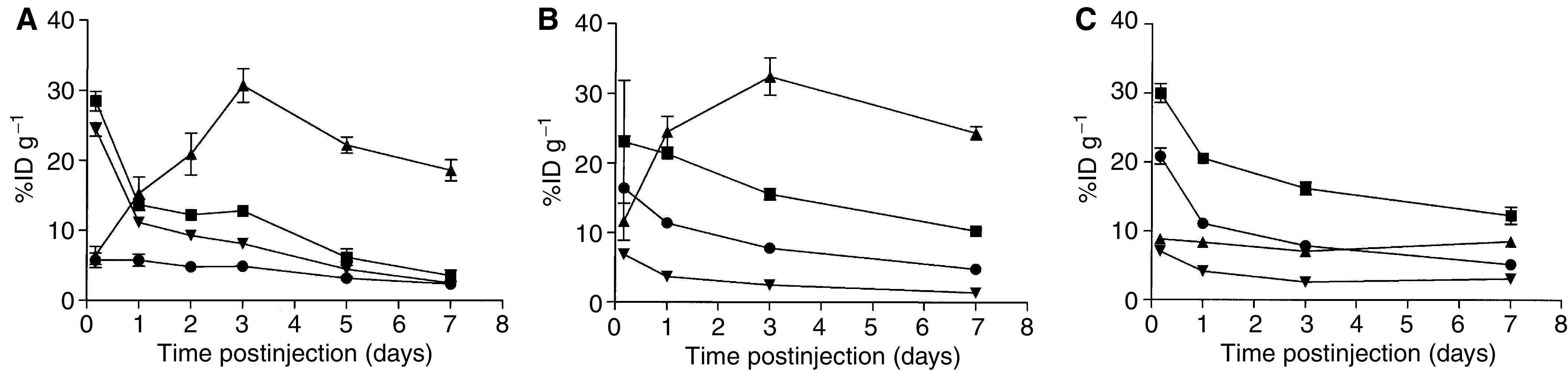
The biodistribution over 7 days of ^111^In-CHX-A″-DTPA- and ^125^I-labelled ch806 in (**A**) U87MG.de2-7 xenografts, (**B**) A431 xenografts, (**C**) FaDu xenografts and serum from BALB/c nude mice. Results are expressed as mean (bars; ±s.d.) percent injected dose per gram (%ID g^−1^) for each time point, (*n*=3–5 mice). The data are presented as: ^111^In-serum (▪); ^111^In-tumour (▴), ^125^I-serum (•), and ^125^I-tumour (▾).

**Figure 4:**
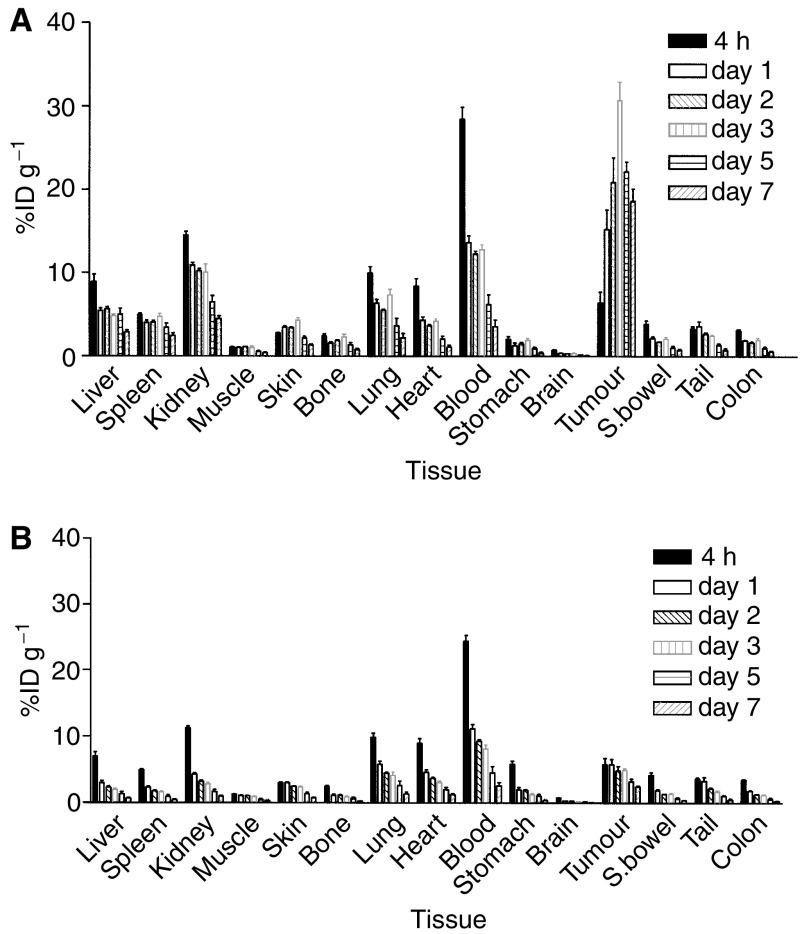
Normal tissue biodistribution of radiolabelled ch806 over 7 days in BALB/c nude mice bearing tumour xenografts (*n*=5). Results of the biodistribution of (**A**) ^111^In-CHX-A″-DTPA-ch806 and (**B**) ^125^I-ch806 are presented for each tissue expressed as mean (bars;±s.d.) percent injected dose per gram (%ID g^−1^) values.

The ^125^I/^111^In-labelled biodistribution study results are presented in Figures 3 and 4. The comparison of %ID g^−1^ of ^I25^I- and ^111^In-labelled ch806 in mice bearing U87MG.de2-7 xenografts are detailed in Figure 3A and in mice bearing A431 tumours in Figure 3B. The %ID g^−1^ of ^l25^I-ch806 in U87MG.de2-7 tumours peaked at 24 h while the ^111^In-ch806 in U87MG.de2-7 xenografts peaked at 48 h following antibody injection with the uptake of ^111^In-labelled ch806 being four-fold greater than ^I25^I (mean: 30.7 *vs* 7.2% ID g^−1^, respectively) and reaching statistical significance (P=<0.001). Uptake of ch806 within glioma xenografts was superior for the ^111^In label at all time points studied, and of note the ^111^In uptake was more than six-fold that of ^125^I at *t*=72 h to 312 h inclusive (Figure 3A).

Despite only binding ∼10% EGFR on A431 cells the ^111^In-ch806 demonstrated a mean peak uptake at 3 days of 33%ID g^−1^ and was highly retained within the tumour over the study period, in marked contrast to ^125^I-ch806, which showed minimal specific A431 tumour uptake. A peak A431 tumour : blood ratio of 5 : 1 was observed at day 7 for ^111^ln-ch806. No specific tumour uptake was observed with either radioconjugate in the FaDu xenografts which express normal tissue levels of EGFR (Figure 3C).

The biodistribution of radiolabelled ch806 in normal murine tissues was studied, and results for time periods between 4 and 312 h following injection of radiolabelled ch806 in mice bearing U87MG.de2-7 xenografts are presented in Figure 4. Highly comparable patterns of biodistribution in normal tissues were observed in mice bearing A431 and FaDu xenografts (data not shown). The %ID g^−1^ in all normal tissues was less than 10% except for the kidney and blood where initial activity was consistent with blood pool of the radioconjugates.

### Pharmacokinetics ch806

Calculation of pharmacokinetic parameters for ch806 are presented in Table 2. Similar times for initial and terminal phases of disposition were observed of the ^111^In- and ^125^I-labelled ch806 conjugates, with the slightly slower clearance of ^111^In-ch806 attributing to a greater AUC for this conjugate.

### Ch806 therapy of established tumour xenografts

**Figure 5:**
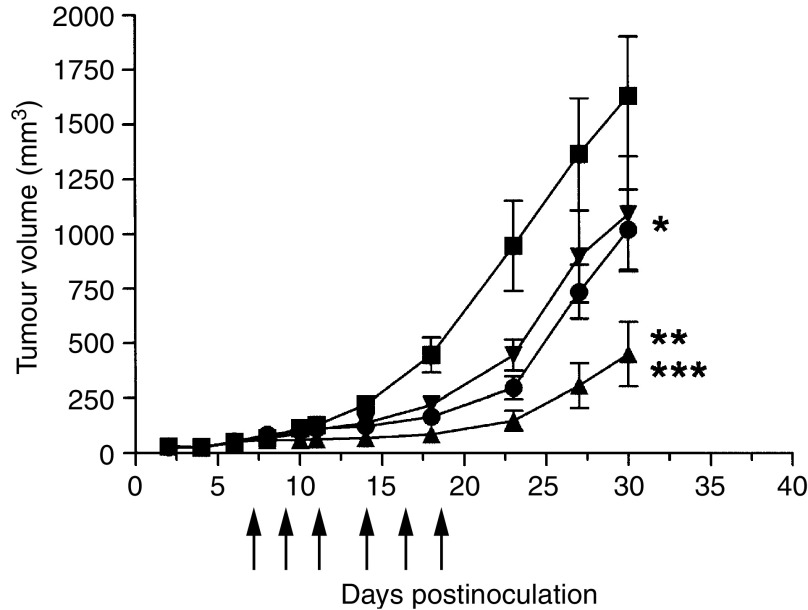
Antitumour effect of ch806 on U87MG.de2-7 glioma xenograft growth rates. U87MG.de2-7 cells (3 × 10^6^) were injected s.c. into both flanks of 4–6-week-old BALB/c nude mice at day 0. Antibody treatment commenced on day 7 post-tumour-cell inoculation and consisted of six i.p. injections over 2 weeks of 1 mg ch806 (▴), 1 mg mAb 806 (•), 1 mg 528 anti-EGFR (▾), or vehicle control (▪) on days 7, 9, 11, 14, 16 and 18 (arrows). Mean tumour volume (bars;±s.e.) for each treatment group (*n*=5) is presented. ^*^P=0.04, mAb 806 *vs* control, day 30. ^**^P=0.001, ch806 *vs* control, day 30. ^***^P=0.014, ch806 *vs* mAb 806, day 30; *P*=0.025, ch806 *vs* 528, day 30.

The results of *in vivo* therapy experiment in BALB/c mice bearing de2-7EGFR-positive xenografts are presented in Figure 5. Compared to vehicle control, the murine mAb 806 significantly inhibited the growth of U87MG.de2-7 gliomas by day 14 (*P*<0.05), and this growth inhibition remained significant up to day 30 (*P*=0.04). The pan anti-EGFR antibody 528 significantly inhibited the growth of U87MG.de2-7 gliomas by day 14 (*P*<0.05); however, this growth inhibition was significant only to day 23 (*P*=0.023). Ch806 showed more significant growth inhibition by day 10 (*P*<0.05), and tumour growth remained significantly inhibited to day 30 (*P*=0.001). Ch806 inhibition of tumour growth was significantly greater than mAb 806 (*P*=0.014) or 528 (*P*=0.025) on day 30. There were 30% complete responders by day 30 for ch806, and none for mAb806 or 528.

## DISCUSSION

We report the successful engineering and expression of a chimeric monoclonal antibody with specificity for the unique epitope bound by mAb 806 found on both de2-7 EGFR and amplified or overexpressed EGFR (Luwor *et al*, 2001; Mishima *et al*, 2001; Johns *et al*, 2002, 2004; Jungbluth *et al*, 2003). Ch806 was stably expressed in CHO cells and exhibited antigen binding specificity and affinity identical to that of its parent murine antibody. These results are not unexpected since the antigen binding variable regions are not altered in the chimeric version and remain murine in origin. Much technological progress has been made in the last decade on the generation of CDR-grafted humanised antibodies and fully human antibodies in an attempt to remove the murine nature of the variable region seen with chimeric antibodies (Clark, 2000). Often the humanisation process results in antibodies where the antigen binding affinity is significantly lower than the murine parent antibody. In our clinical experience, we have not seen greater immunogenicity for chimeric antibodies compared to humanised antibodies (Ritter *et al*, 2001; Scott *et al*, 2001, 2003). In fact, taken together with the cost of generating a humanised antibody (including intellectual property and time for generation) and the often impaired antigen binding activity, we believe chimeric antibodies are a suitable format for translation of a murine antibody with potential into a human product candidate. This is reflected in the scope of chimeric monoclonal antibodies in the clinic, and the FDA's approval for ReoPro® (abciximab), Rituxan® (rituximab), Remicade® (infliximab), Simulect® (basiliximab) and Erbitux® (cetuximab).

In contrast to the parental murine mAb 806 immune effector function, the chimerisation process led to an antibody with strong ADCC activity. Interestingly, there was no concurrent increase in CDC activity. This immune effector function profile for chimeric antibodies generated in DG44 cell has been observed with a number of antibodies generated within our laboratory (Panousis, personal communication). It is possible that the glycosylation profile of CHO-cell-derived antibodies impairs or prevents the binding of Clq and consequent activation of the classical complement pathway (White *et al*, 1997). This phenomenon is being explored further in our laboratory. The levels of ADCC achieved with the two different target cell lines reflected the number of ch806 binding sites on the two cell populations. Ch806 targets ∼1 × 10^6^ de2-7EGFR on U87MG.de2-7 cells effecting greater ADCC compared to A431 cells where only a subset of the 2 × 10^6^ wt EGFR molecules are recognised by ch806 (Luwor *et al*, 2001; Johns *et al*, 2002). The strong ADCC activity displayed by ch806 compared to the murine antibody (which lacks significant ADCC) has provided a further tumour cell killing mechanism to enhance the direct signalling-mediated killing activity displayed by ch806.

We have examined the *in vivo* biodistribution, pharmacokinetics and labelling stability of ch806. ^125^I- and ^111^In-conjugated ch806 retained binding affinity to de2-7EGFR-expressing glioma cells and were also stable *in vitro* as assessed by ITLC. The binding affinity of ch806 was retained, despite prolonged incubation at 37°C in serum and during *in vivo* animal experiments. Pharmacokinetic and biodistribution studies in BALB/c nude mice demonstrated a long serum half-life and excellent and specific targeting properties for tumours containing de2-7EGFR or amplified *EGFR.* The prolonged and superior tumour uptake observed for ^111^In-ch806 compared to the ^l25^I-conjugate suggest that ch806 is rapidly internalised following binding with concomitant catabolism and excretion of the radiohalide from the tumour cell. Other xenograft studies with internalising antibodies have also demonstrated lower absolute levels of iodine uptake compared with ^111^In (Clarke *et al*, 2000). The differences in tumour retention of radiolabel in this study most likely represents differences in cellular processing of radiolabelled ^I25^I as opposed to ^111^In. The current observation is consistent with the rapid internalisation characteristics of mAb 806 observed in confocal and EM experiments with de2-7 and amplified EGFR-expressing cell lines (Johns *et al*, 2002). The need to utilise ^111^In to visualise antibody tumour localisation raises the issue of how many antibodies have been prematurely aborted from further development based on initial results from biodistribution studies undertaken using radio-iodine-labelling and suggests that negative results obtained thusly should always be confirmed with a radiometal-based nuclide.

The ability of ch806 to inhibit the growth of de2-7-expressing tumour xenografts in BALB/c mice was compared to the murine parent antibody and the anti-EGFR antibody 528. While mAb 806 demonstrated similar tumour growth inhibition to 528, the results clearly show that the chimerisation process has improved the ability of ch806 to impair significantly the growth of tumour xenografts *in vivo.* A direct killing mechanism impacting on cellular signalling mechanisms required for tumour cell proliferation remains a major factor in the likely mode of action for ch806; however, some immunological function using murine effector cells (ADCC) may also account for the improved efficacy of ch806 over mAb 806. Mechanisms associated with the anti-tumour activity of ch806 are under further investigation in our laboratory.

The overexpression of EGFR in many types of epithelial cancers EGFR renders it an attractive target for tumour-targeted antibody therapy. A number of EGFR antibodies have been reported in the literature with several undergoing clinical evaluation. (Arteaga, 2002; Herbst and Langer, 2002) and more recently the chimeric antibody C225 (Erbitux®) was approved for use with irinotecan in advanced colon cancer. The antitumour activity of most EGFR antibodies, including the codeveloped 225 and 528, is enhanced by their ability to block ligand binding (Arteaga, 2002; Herbst and Langer, 2002). Such antibodies may mediate their efficacy through both modulation of cellular proliferation and antibody-dependent immune functions (e.g. complement activation). The mAb 806 was generated following immunisation with mouse fibroblasts expressing the de2-7 EGFR and was selected by mixed haemadsorption assay for high reactivity to de2-7 EGFR and negligible activity against the wt EGFR (Jungbluth *et al*, 2003). Further characterisation revealed that mAb 806 could recognise cell lines and glioma specimens when the wt EGFR was overexpressed, especially when the *EGFR* gene was amplified, but not normal tissue (Johns *et al*, 2002). Our recent observation that mAb 806 binds an epitope revealed by the de2-7 EGFR truncation that is also exposed by the untethered EGFR, suggests that 806 prevents the formation of signalling-capable EGFR (Johns *et al*, 2004). The chimerisation of 806 has markedly improved the ADCC effected by this antibody and the *in vivo* characteristics of this antibody show great promise for clinical studies.

In conclusion, we have generated a chimeric antibody with specificity for the de2-7 EGFR and amplified EGFR, but not normal tissue, by targeting a novel epitope that is only transiently exposed. The tumour-restricted expression and cell surface location of the 806 antigen make it an ideal target for immunotherapeutic strategies utilising ch806 with exciting clinical potential against a variety of human epithelial tumours including head and neck tumours and glioma. In contrast to other anti-EGFR antibodies in clinical development or approved by the FDA (e.g. Erbitux®) that also bind normal EGFR present on normal human skin and liver, ch806 is a novel direct-killing anticancer antibody with specificity only for the tumour it is targeting, and not normal human cells. This provides opportunities for naked antibody therapy, radioimmunotherapy and the use of ch806-based drug immunoconjugates, where a cytotoxic agent could be used to target the tumour and possibly work synergistically with the direct killing mechanisms of ch806, yet sparing toxicity to normal cells. The *in vitro* characteristics and stability of ch806 together with the *in vivo* biodistribution and antitumour data indicate properties that are suited for translation to the clinic in Phase I trials.

## Figures and Tables

**Table 1:** Radiolabelled ch806 serum stability at 37°C

**Time (day)**	**0**	**4**	**7**
^111^In-ch806 radio purity (%)	99.5	97.4	96.9
^111^In-ch806 IR (%)	69.5	63.7	66.7
^125^In-ch806 radio purity (%)	99.0	95.6	95.0
^125^In-ch806 IR (%)	68.1	62.2	63.2

**Table 2:** Pharmacokinetic parameters for radiolabelled ch806 in serum and tumour (mean±s.e.)

	***T*_1/2α_ (h)**	***T*_1/2β_ (hr)**	**AUC (h *μ*g ml^−1^)**	**CL (ml h^−1^)**
Serum ^111^In-ch806	3.42±0.92	78.64±15.40	66.79±7.94	0.045±0.005
Serum ^125^I-ch806	2.85±0.24	69.87±6.56	47.52±2.61	0.063±0.003
